# Application of Biomineralization Technology to Self-Healing of Fiber-Reinforced Lightweight Concrete after Exposure to High Temperatures

**DOI:** 10.3390/ma15217796

**Published:** 2022-11-04

**Authors:** How-Ji Chen, Hsien-Liang Chang, Chao-Wei Tang, Ting-Yi Yang

**Affiliations:** 1 Department of Civil Engineering, National Chung-Hsing University, 145 Xingda Rd., South District, Taichung City 402, Taiwan; 2 Department of Civil Engineering and Geomatics, Cheng Shiu University, No. 840, Chengching Rd., Niaosong District, Kaohsiung 83347, Taiwan; 3 Center for Environmental Toxin and Emerging-Contaminant Research, Cheng Shiu University, No. 840, Chengching Rd., Niaosong District, Kaohsiung 83347, Taiwan; 4 Super Micro Mass Research and Technology Center, Cheng Shiu University, No. 840, Chengching Rd., Niaosong District, Kaohsiung 83347, Taiwan

**Keywords:** biomineralization, high temperatures, fiber reinforced lightweight aggregate concrete, residual mechanical properties

## Abstract

In the field of civil engineering, concrete self-healing technology plays an important role. Concrete self-healing should be able to effectively heal cracks, not only improving the internal structure, but also improving the mechanical properties and durability of the concrete structure. The biomineralization-repair method is characterized by its potential for long-lasting, rapid, and active crack repair potential. Biomineralization repair has an effective bond ability, is compatible with concrete components, and is also environmentally friendly. This study used biomineralization to explore the self-healing of fiber-reinforced lightweight concrete after its exposure to high temperatures. Concrete specimens of a control group (using lightweight aggregate without bacterial spores and a nutrient source) and an experimental group (using lightweight aggregate containing bacterial spores and a nutrient source) were prepared. The repair effect of the microbial self-healing concrete after the exposure to high temperature was observed by a crack-width gauge, field-emission scanning electron microscopy (FESEM), energy-dispersive spectroscopy (EDS), and X-ray diffraction (XRD). According to the EDS and XRD analyses, the precipitate formed at the crack was calcium carbonate. After 28 days of self-healing, the water absorption rate of the experimental group was lower than that of the control group. This is because the specimens of the penetration test were taken from the middle of the concrete cylinder after high temperature, and their bacterial survival rate was higher, which made the mineralization more significant. However, the mechanical test results of the control and experimental groups after the self-healing in the water were not substantially different, which indicated that the bacterial mineralization in the experimental group was slow in the absence of an adequate source of nutrients.

## 1. Introduction

Concrete, which is the most used civil construction material today, has the advantages of low material costs and maintenance requirements [1]. Due to the influence of the external environment, overload or accidental damage, high-temperature fire damage, and other factors, concrete often cracks or is locally damaged, which adversely affects its impermeability and chloride-ion corrosion and carbonization resistance, which reduces the durability and service life of concrete structures [2]. In view of this, the focus of the research by the experts in the field of concrete all over the world has been on repairing concrete cracks in time to prolong the lives of concrete structures. However, the repair of concrete structures is expensive and requires frequent maintenance. In general remediation, commercially available chemicals and polymers are used, which are a source of health and environmental risks. Furthermore, they only work for a short period of time. Therefore, many researchers have been committed to the development of ecologically harmless and sustainable biological-system repair [3,4,5,6,7,8,9,10,11,12,13,14,15,16,17,18,19,20,21,22].

In the field of concrete science, the systems of self-healing concrete are mainly divided into two types: autogenic and autonomic [4]. The self-healing of concrete mainly depends on its composition [5]. The autogenous healing of concrete is a traditional inherent material-repair characteristic, and its self-healing process starts from the existing general materials [3]. For example, the self-healing ability exhibited by cementitious materials can protect concrete from damage due to the rehydration properties of the unhydrated cement that remains on the crack surface [6]. In a concrete matrix, about 15–25% of the cement is in an unhydrated form. Once cracking occurs, these bare and unhydrated cement particles will begin to absorb water and their hydration processes will begin, thereby filling and healing the crack. The delivery of water may come from the natural environment, or it may be provided by man-made means. Many researchers have pointed out [3,4,5,6,7,8,9,10,11,12,13,14,15,16] that, through the secondary hydration of the unhydrated cement around the cracks and the precipitation of calcium hydroxide crystals, or other physical and mechanical behaviors, concrete microscopic cracks can repair themselves to a certain extent [5]. Autogenous healing has some benefits, but only for very narrow fissures [9].

Autonomous self-healing is a self-healing process that combines material components that are not traditionally used in concrete. In contrast to autogenous healing, autonomous self-healing in concrete requires the release of a healing agent from a prepared capsule or continuous network. In this field, microbial methods have attracted a lot of attention [17], and the term “microbe” refers to many different types of organisms. To date, the research work on autonomous self-healing concrete has mostly been limited to bacteria [18]. Researchers have extensively studied bacteria-mediated self-healing concrete based on the bioinduced mineralization process [19,20], and they have shown that bacteria are able to precipitate calcium carbonate that is highly compatible with concrete components [11]. Biomineralization is a natural effect defined as the process by which organisms produce minerals through metabolic activities associated with environmental interactions [15]. During biomineralization, organisms utilize biopolymers to generate biomineral phases [21]. Metabolites secreted by microorganisms react with ions or compounds in the environment, thereby contributing to subsequent mineral particle changes that develop into metabolite deposition [22]. Over the past two decades, the application of microbiologically induced calcium carbonate precipitation (MICP) in concrete self-healing has been a focus of the concrete academic community [3,4,5,6,7,8,9,10,11,12,13,14,15,16,17,18,19,20,21,22]. The biomineralization reaction is mild, and the resulting substance is calcium carbonate (CaCO_3_), which itself is a natural stone. Therefore, biomineralization is not only environmentally friendly and durable, but it is also compatible with cement-based materials [23].

Based on the abovementioned research, the self-healing of concrete should be able to effectively heal its cracks, not only improving the internal structure, but also improving the mechanical properties and durability of the concrete structure. The microbial-mineralization-remediation methods are characterized by their potential for durable, rapid, and active fracture repair. The methods have effective bonding capacities, are compatible with concrete compositions, and are also environmentally friendly. Fire damage caused by high temperatures is a special failure mode of concrete structures. The repair of concrete after exposure to high temperatures has a huge impact on the overall safety of the structure, which has great research significance and value. So far, the research on the long-term durability and residual mechanical properties of concrete repaired by microbial mineralization after exposure to high temperatures, and this needs to be further explored. In view of this, this study used biomineralization to explore the self-healing of fiber-reinforced lightweight concrete after its exposure to a high temperature from the perspective of sustainable development. The effect of the microbial self-healing of concrete cracks after exposure to high temperature was studied by the crack-width gauge, field-emission scanning electron microscopy (FESEM), energy-dispersive spectroscopy (EDS), and X-ray diffraction (XRD).

## 2. Experimental Procedure

### 2.1. Experimental Program

When researchers perform the bacterial-liquid-fermentation process of MICP in the laboratory, the important factors are the composition of the nutrient solution, oxygen input, temperature control, the stirring rate, and environmental control. For the prepared bacterial culture medium, the concentration of bacterial liquid was observed by a visible light spectrometer to understand the growth of the microorganisms. In addition, the temperature-treated Bacillus nutrient solution was observed by field-emission scanning electron microscopy (FESEM), and the spore-staining method was used to verify the spores. After that, a urease test was performed. On the other hand, the mixing of the proportions of the concrete and the casting of the specimens were carried out. The test items included a water-penetration test, compressive-strength test, flexural-strength test, observation of the crack repair after the exposure to a high temperature, FESEM observation, and EDS and XRD analyses. After curing to 28 days of age, some of the specimens were subjected to a water-penetration test, compressive-strength test, and flexural-strength test at room temperature. While some of the specimens were first subjected to a high temperature test, and then they were immersed in the curing tank of the laboratory. After self-healing to the planned age, the crack-healing observation and the water-penetration, compressive-strength, and flexural-strength tests, as well as the repair-compound identification were carried out. 

### 2.2. Materials

**Figure 1:**
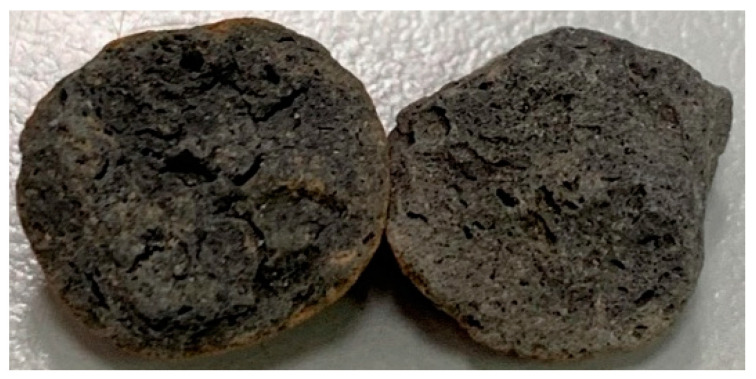
Appearance of lightweight expanded shale aggregates.

**Figure 2:**
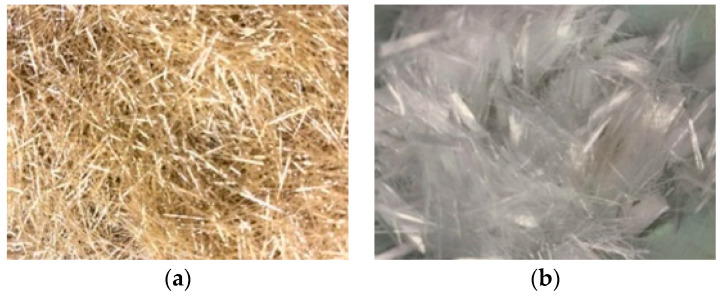
Fibers used in this study: (**a**) short micro-steel fibers; (**b**) polypropylene fibers.

The materials used in this study can be divided into two parts: the bacterial culture and concrete (calcium lactate as a nutrient source for *Bacillus pasteurii*):

- *Bacillus pasteurii* (DSM 33): A Gram-positive aerobic bacterium that is ubiquitous in soil and can produce a large amount of intracellular urease. It was ordered from the Biological Resource Conservation and Research Center of the Food Industry Development Research Institute (strain number: BCRC11596);
- Calcium lactate: It was used as a source of nutrients for the *Bacillus pasteurii*, it iwas embedded in the lightweight aggregates;
- Yeast extract (YE): YE is used as a nutrient for bacteria, which contains vitamins, minerals, amino acids, etc.; its leaching solution is used as a culture medium;
- Calcium acetate: It was used as a source of the calcium ions for biomineralization during concrete self-healing;
- Urea: It was used as a source of the carbonate ions for biomineralization during concrete self-healing;
- Cement: A locally produced Portland Type I cement with a specific gravity of 3.15 and a fineness of 3400 cm^2^/g was used;
- Water: It was general tap water, which is in line with the quality requirements of concrete mixing water;
- Fine aggregate: It was a locally produced natural river sand, with a specific gravity of 2.6, a water-absorption rate of 2%, and a fineness modulus of 2.7;
- Lightweight aggregate: The lightweight coarse aggregate was artificial aggregate, as shown in Figure 1. The original maximum particle size is 3/4 inches; the crushed maximum particle size is 3/8 inches; the particle density is 1.57 g/cm^3^; the dry unit weight is 927 kg/m^3^; the specific gravity is 2.65; the water absorption is 6%; the crushing strength is 12.67 MPa;
- Superplasticizer: Its code name is R-550, and it is a product of the Taiwan Sika Company; its chemical composition is water-modified polycarboxylate; it meets the requirements of ASTM C494-81 Type F;
- Fibers: These were the products of Guli Li Co., Ltd. We used short microsteel fibers (according to ASTM A820) and polypropylene fibers, as shown in Figure 2. The basic properties of the two fibers is shown in Table 1.

### 2.3. Culture of Bacterial Strains and Sporulation

In terms of the sterilization of the bacterial culture containers and equipment, we first sealed the conical flask with aluminum foil, a sponge plug, or special iron cover, and we then put the microplastic straw tip in the special container. After that, we sealed the graduated cylinder with aluminum foil, slightly opened the lid of the centrifuge tube and placed the sterilization bag inside, placed the microcentrifuge tube in a special container, covered the inoculum ring and bacteria-coated stick with aluminum foil, and sterilized it in an autoclave (121 °C; 1.5 kg/cm^2^; 20 min), followed by drying. In terms of configuring the liquid culture medium, the steps are as follows:

(a) Count the medicines and prepare clean containers (beakers, Erlenmeyer flasks, graduated cylinders);
(b) Separately prepare the medicines according to the different sterilization methods (those that may be decomposed by heat must be sterilized by filtration, and the rest should be sterilized by high-pressure steam);
(c) The medicine sterilized by high-pressure steam should be cooled at room temperature and then added to the filter sterilization;
(d) Liquid-medium formula:
  - Yeast extract: 20 g/L;
  - TRIS-hydrochloride (NH_2_C(CH_2_OH)_3_·HCl): 20.488 g/L;
  - Ammonium sulfate (NH_4_SO_4_): 10 g/L;
  - Sodium hydroxide (NaOH) (adjust pH above 9).

In terms of the culturing bacteria, the details are as follows:

(a) After cooling the liquid medium to room temperature, dispense the liquid medium into the bacteria-culturing containers (Erlenmeyer flasks, centrifuge tubes, or test tubes) on a dust-free sterile operating table;
(b) Inoculation strain (*Bacillus pasteurii*): Use a micropipette to add the bacterial solution to the liquid medium and shake well;
(c) Place in a constant-temperature rotary-shaking incubator at 37 °C with a rotating speed of 180 rmp for the suspension culture on a shaker, and culture in batches from three to four days;
(d) Use a visible-light spectrometer to observe the concentration of bacterial liquid (that is, measure the optical density (OD)). Observe and record the OD_600_ value (that is, the spectral absorption value at a wavelength of 600 nm) to about 1.2, and then end the bacterial culture.

### 2.4. Strain Implantation

**Figure 3:**
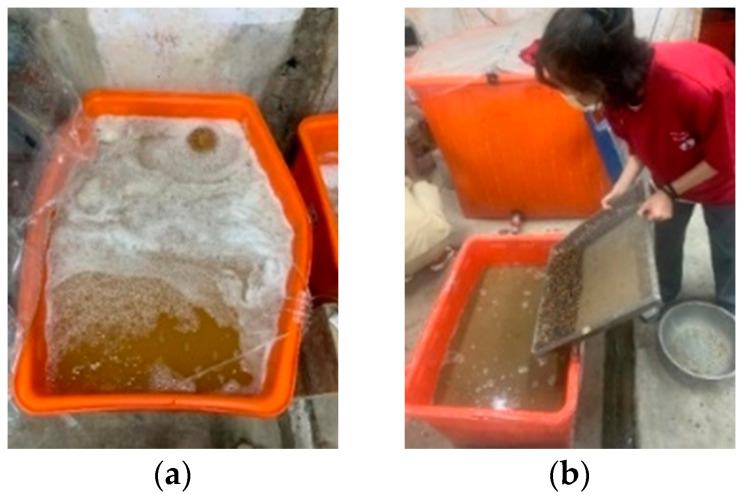
Strain implanted in lightweight aggregate: (**a**) bacterial spore solution; (**b**) lightweight aggregate dipped in bacterial spore solution.

**Figure 4:**
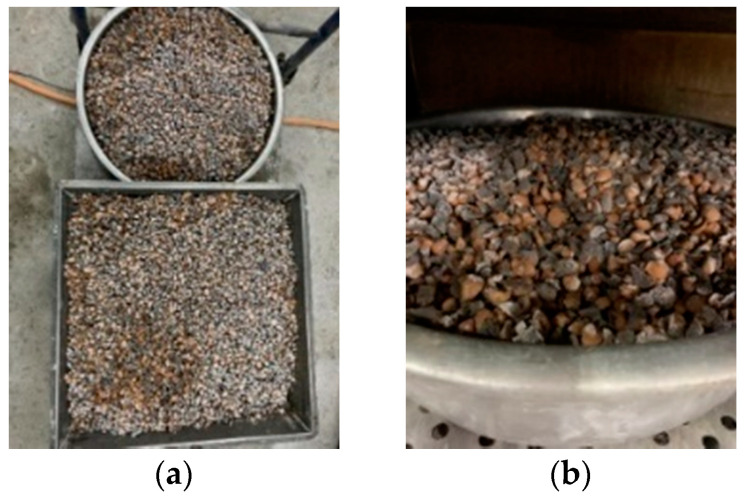
Draining and drying of lightweight aggregates: (**a**) draining; (**b**) drying.

In this study, the lightweight aggregate was used as the bacterial carrier, and then the lightweight aggregate was mixed into the concrete to increase the survival probability of the bacteria. When the external environment meets the growth conditions of bacteria, the vitality of the strain can be restarted. To this end, the strain was sporulated by simulating a severe environment by increasing the temperature. The cultured bacterial liquid was first placed in a constant temperature water tank, the temperature of the water tank was set to 80 °C, and the temperature holding time was set to 30 min. Then, the bacterial liquid at a temperature of 80 °C was taken out, cooled at room temperature, and its OD_600_ value was measured. As for the bacterial liquid used to soak the lightweight material, the clear liquid part will be taken. The steps to implant the strain into the lightweight aggregates are as follows:

(a) The lightweight aggregates were immersed in a nutrient source solution containing calcium lactate (80 g/L) and yeast extract (1 g/L) for 30 min, as shown in Figure 3.
(b) Drain the lightweight aggregates that were soaked in the nutrient-source solution, and then put them into an oven at 37 °C to dry for 5 days, as shown in Figure 4.
(c) Repeat the previous two steps once; then, immerse the nutrient-containing lightweight aggregate in the bacterial spore solution for 30 min; and
(d) Drain the lightweight aggregates soaked in the bacteria-spore solution and place them in an oven at 37 °C for 5 days. After that, the preparations for the implantation of the strains in the lightweight aggregates were completed.

### 2.5. Mix Proportions of Concrete and Casting of Specimens

In accordance with the process of ACI 211.2, Standard Practice for Selecting Proportions for Structural. Lightweight Concrete, the amount of each component of the lightweight aggregates concrete is determined according to the material properties and structural performance requirements. To understand the effectiveness of MICP, two groups of concrete were prepared in this study. One group was the concrete using the lightweight aggregate without bacterial spores and nutrients as the control group, and the other group was the concrete using the lightweight aggregate containing the bacterial spores and nutrients as the experimental group. Under the premise of not affecting the workability of concrete, the amount of steel fiber was 0.75% (volume percentage), and the amount of polypropylene fiber was 0.09% (volume percentage). According to the above plan, the concrete mix design of each group is shown in Table 2.

The mixing of the concrete mixture was carried out using a two-shaft electric mixer. The fine aggregates (in an absolutely dry state) and cement were first placed in the mixing barrel, and fully dry-mixed for about one minute at a low speed of 140 ± 5 rpm. At the same time, the steel fibers and polypropylene fibers were evenly dispersed by manual methods, and fully dry mixed for a few minutes until they were in a uniform state. After that, the lightweight aggregates (in a dry state) were placed into the mixing barrel and mixed at a medium speed of 285 ± 10 rpm for about one minute. Then, the pre-mixed water and superplasticizer were slowly poured into the mixing barrel and mixed thoroughly until a homogeneous fresh concrete was formed.

After the mixing of each group is completed, the slump is measured and recorded. Then, according to the relevant regulations of the ASTM and CNS, twelve cylindrical specimens with a diameter of 100 mm and a height of 200 mm, twelve cylindrical specimens with a diameter of 150 mm and a height of 300 mm, and six prismatic specimens with a length of 360 mm, a width of 100 mm and a thickness of 100 mm were cast for each group of concrete. The specimens were demolded after 24 h, and then placed in a water tank for curing. The specimens were not removed from the water tank until 27 days later for the mechanical-property and high-temperature tests.

### 2.6. Test Methods and Data Analysis

The cylindrical specimens were tested for their compressive strengths according to ASTM C39, and for their elastic moduli according to ASTM C469. The compressive strength and elastic modulus were the average values of three specimens. The prismatic specimens were tested for their flexural strengths according to ASTM C78. The prismatic specimen was simply supported and loaded in a three-point mode, and the deflection at the midpoint of its net span was measured at the same time. The flexural strength was the average values of two specimens.

**Figure 5:**
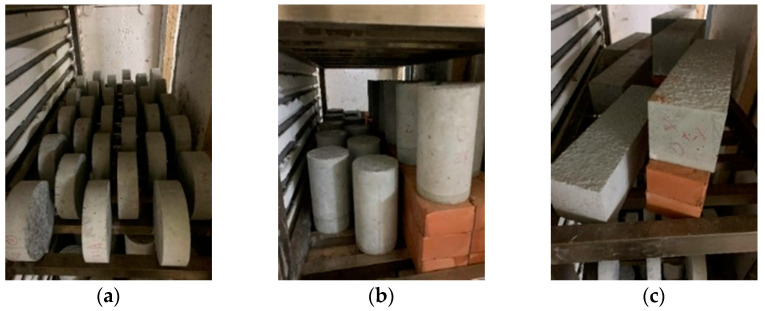
Configurations of specimens in high-temperature furnace: (**a**) water-penetration-test specimens; (**b**) compressive-strength-test specimens; (**c**) flexural-strength-test specimens.

**Figure 6:**
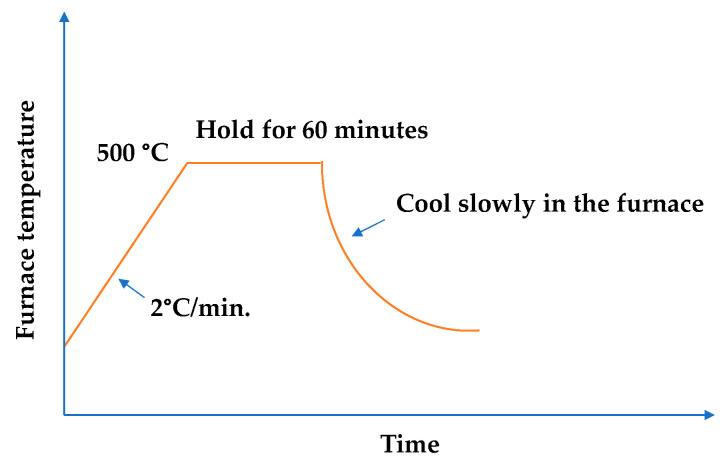
Heating process of the high-temperature test.

The target temperature of the high temperature test was 500 °C. The concrete specimens were placed in a high-temperature furnace with a gap of 20 mm between the specimens, as shown in Figure 5. During the high temperature test, the furnace temperature heating rate was 2 °C/min. After reaching the target temperature of 500 °C, this temperature was continued for one hour, as shown in Figure 6. After that, the specimens were naturally cooled in the high-temperature furnace to a normal temperature of 23 °C, and then the subsequent related tests were carried out. In terms of the microscopic observation, the whole crack was taken out with a cutting machine. The specimens were split along the cracks, and one of the crack sections was taken and placed under a scanning electron microscope to observe the distribution of the generated substances along the crack-depth direction. Then, another section was taken and grounded into the matrix along the fracture surface with a file, and the ground powder was collected and subjected to XRD analysis to understand its composition.

According to the different states of the specimens, the compressive strength and flexural strength tests of the two groups of concrete are divided into four types, and the test sequences are shown in Table 3 and Table 4, respectively. The description of the specimen designation is as follows. The first letter: R means room temperature, H means the sample has been subjected to high temperature; the second letter: C means compression test, F means bending test; the third letter: A means control group, B means experimental group: numbers indicate age of self-healing. # means reload.

**Figure 7:**
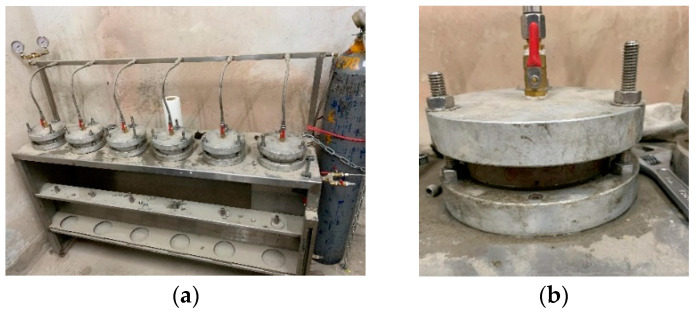
Water penetration test setup: (**a**) the case of six specimens; (**b**) the case of a single specimen.

Each group of concrete was subjected to a water penetration test, according to CNS 3763, before and after exposure to a high temperature. The test configuration is shown in Figure 7. The test steps are as follows:

(a) After cutting the upper and lower sections of the specimen by 7 cm with a cutting machine, take the remaining middle section and cut it into three pieces, making each section 4 cm;
(b) Put the sheet specimen in an oven and dry it at 80 °C for three days;
(c) One hour before the water-penetration test, take the specimen out of the oven and measure the weight before the water penetration;
(d) Dry the water-penetration-test mold up and down, and apply oil on the rubber pad;
(e) Put the specimen into the mold, lock the bolt, open the pressure valve, and apply 3 kgf/cm^2^ of water pressure for three hours;
(f) Remove the specimen, wipe the surface-water stains dry, and record the weight after the water penetration.

The water absorption can be obtained by using the weight of the specimen after the water penetration ($m_{d}$) and the weight before the water penetration ($m_{a}$), and its calculation formula is as follows:

(1) $$\mathrm{Water}\;\mathrm{absorption}=\frac{m_{a}-m_{d}}{m_{d}}\times 100\%$$

The water-absorption rate is the average of six samples.

According to the different states of the specimens, the water penetration tests of the two groups of concrete are divided into three types, and the test sequences are shown in Table 5. Taking Specimen No. RPA0 as an example, the first letter R means room temperature, the second letter P means water penetration test, the third letter: A means control group, B means experimental group, 0 means self-healing age. As for HPA28, the first letter H indicates that the specimen has been subjected to high temperature, and the rest are the same as the previous definitions.

## 3. Experimental Results and Discussions

### 3.1. Results of Fresh Properties Test

The slump, slump flow, and unit weight of each series of concrete are shown in Table 6. The slumps of both the control group and experimental group were 6 cm, and both had proper workability. At the same time, the slump flows of the control group and experimental group were 20 cm. The unit weight of both groups of concrete was 1909.5 kg/m^3^.

### 3.2. Viable Bacterial Cells after Exposure to 500 °C

**Figure 8:**
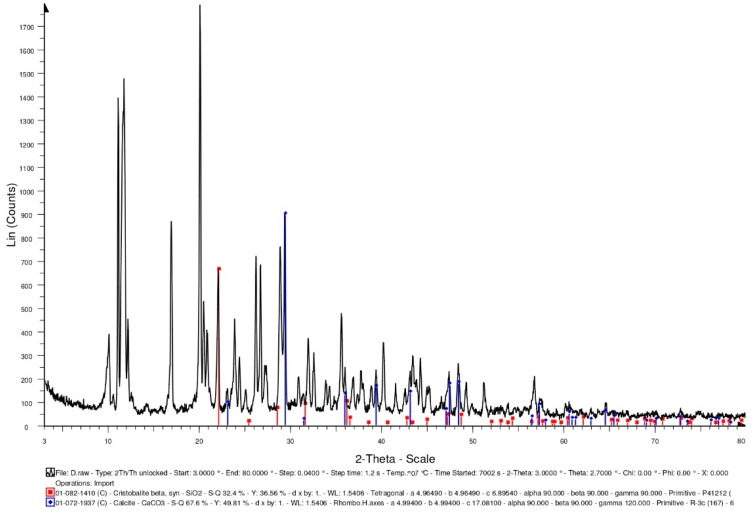
XRD-analysis results of the bacteria powders not subjected to high temperature.

Generally speaking, bacterial cells do not easily survive in high-temperature environments (above 135 °C). In the concrete specimens of the experimental group, the light aggregates carried the bacterial cells. The survival of the bacteria needs to be explored after 500 °C. In this study, the bacterial solution was added to the calcium source solution (calcium lactate: 80 g/L; urea: 20.02 g/L). After the complete reaction, it was dried in an oven (50 °C) until powdery. The intensity of the X-ray-reflected energy of the calcium carbonate in the bacterial powder not subjected to a high temperature was 900 (unit: counts) at 2-theta of 29.4° (Figure 8).

**Figure 9:**
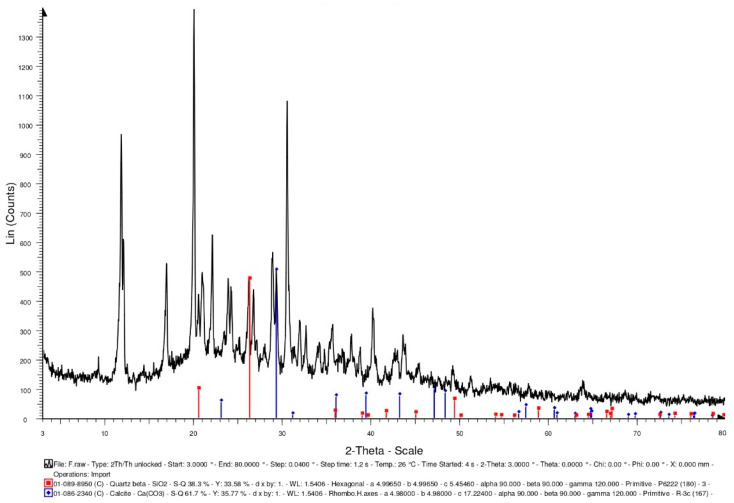
XRD-analysis results of the bacteria powders subjected to high temperature.

**Figure 10:**
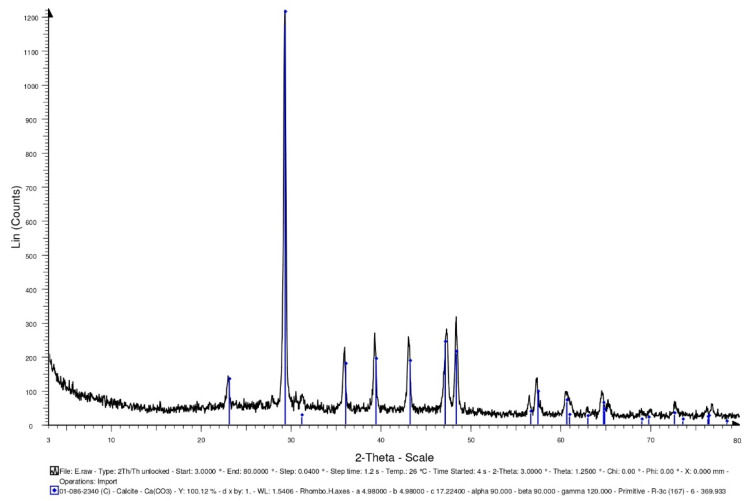
XRD-analysis results of the bacteria powders subjected to high temperature (using lightweight aggregates as a carrier).

Furthermore, some of the bacterial liquids were taken out separately and placed in a high temperature furnace. At a heating rate of 2 °C/min, the temperature was raised to 500 °C, and the temperature was maintained for one hour. After the high temperature test was completed and the temperature was lowered to room temperature, the bacteria liquid subjected to a high temperature was recultivated. The cultured bacterial solution contained the calcium source solution (calcium lactate: 80 g/L; urea: 20.02 g/L). After the complete reaction, it was dried in an oven (50 °C) until powdery. The intensity of the X-ray-reflected energy of the calcium carbonate in the bacterial powder subjected to a high temperature was 510 (unit: counts) at 2-theta of 29.4° (Figure 9). According to this result, there was a reduction in the viable bacterial cells after the exposure to 500 °C, which may have resulted in less pronounced mineralization. On the other hand, the lightweight aggregates were soaked in the bacterial liquid. After the lightweight aggregates were drained, they were placed in a high-temperature furnace. At a heating rate of 2 °C/min, the temperature was raised to 500 °C, and the temperature was maintained for one hour. After the high temperature test was completed and the temperature was lowered to room temperature, the bacteria liquid subjected to the high temperature was recultivated. The cultured bacterial solution contained the calcium source solution (calcium lactate: 80 g/L; urea: 20.02 g/L). After the complete reaction, it was dried in an oven (50 °C) until powdery. When using the lightweight aggregates as carriers, the intensity of the X-ray-reflected energy of the calcium carbonate in the bacterial powder subjected to the high temperature was 1220 (unit: counts) at 29.4° (Figure 10). According to the results, using lightweight aggregates as carriers, after exposure to 500 °C, the bacterial solution was recultivated, and its mineralization was still significant.

### 3.3. Repair-Compound Identification

#### 3.3.1. FESEM Observation and EDS Analysis after 14 Days of Self-Healing

**Figure 11:**
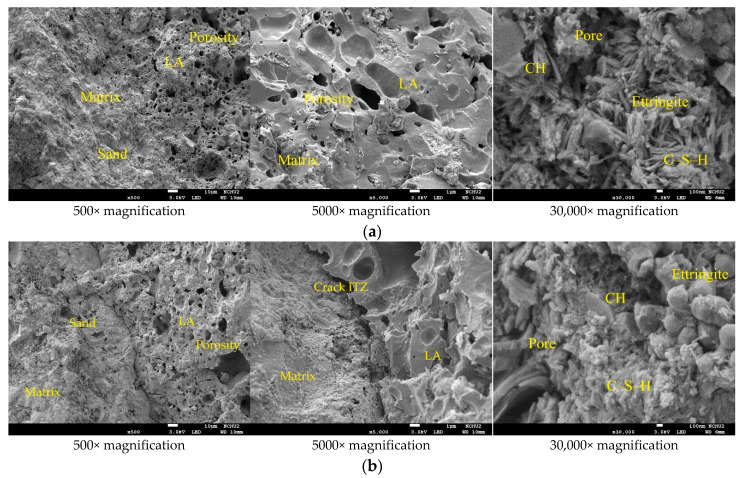
SEM micrographs of the concrete specimens after self-healing for 14 days: (**a**) control group; (**b**) experimental group.

The FESEM micrographs of the concrete specimens after self-healing for 14 days is shown in Figure 11, which reveal information about the pore structures, cracks, and mineral phases of the specimens. In Figure 11, the unhydrated cement particles, matrix, light aggregates (LAs), fine aggregates, and the interface transition zone (ITZ) between the matrix and LAs could be observed. In addition, irregular particles, micropores, and microcracks were also observed in each group of specimens. The higher magnification of Figure 11 revealed a more or less connected network of needle-like and platelet-like crystals, with the elongated hexagonal needles being Ettringite (AFt), and the hexagonal platelets being calcium hydroxide (CH). In addition, the network flocs were calcium-silicate-hydrate (C-S-H) colloids, and the black parts were the pores of the substrate, lightweight aggregates, and ITZ. Among them, C-S-H colloids are the main hydration product of Portland cement, accounting for about 2/3 of the total hydration product in hardened cement slurry, and dominating the macroscopic properties of cement-based materials [2]. Furthermore, many microscopic pores were found inside the lightweight aggregates. In particular, the porosity and pore size distribution are the main factors that control the strength of concrete [2]. In the concrete specimens, the unhydrated silicate was hydrated into calcite. In other words, in the presence of moisture, the carbonation process produced smaller rehydration products [24]. These products reduced the porosity of the concrete and restored some of its strength.

**Figure 12:**
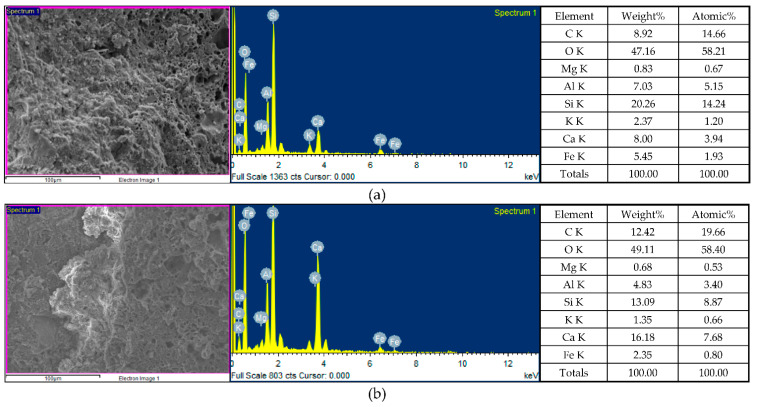
EDS analysis of the concrete specimens after self-healing for 14 days: (**a**) control group; (**b**) experimental group.

After self-healing for 14 days, the concrete specimens were analyzed by EDS for the weight percentages of the various chemical elements. The EDS spectra of the concrete specimens are shown in Figure 12, in which the y-axis describes the number of X-rays, and the x-axis describes the energies of the X-rays. In addition, the position of the peak is the identification of the element, and the peak height helps to quantify the content of each element in the specimens. According to the EDS analysis of the control-group specimens, they contained the elements C, O, Mg, Al, Si, K, Ca, and Fe (Figure 12a), of which the main elements (weight percentage) were O (47.16%), Si (20.26%), C (8.92%), and Ca (8.0%). According to the EDS analysis of the experimental-group specimens, they contained the elements C, O, Mg, Al, Si, K, Ca, and Fe (Figure 12b), of which the main elements (weight percentage) were O (49.11%), Ca (16.18%), Si (13.09%), and C (12.42%). According to the EDS analysis (Figure 12), bacterial CaCO_3_ crystals were present in the experimental-group specimens. In other words, the EDS verified the formation of calcium carbonate in the experimental-group specimens. Moreover, it was found that a higher percentage of elemental calcium in the experimental-group specimens. This may be caused by the mineralization of bacteria. An important stoichiometric parameter that defines the C-S-H phase is the atomic ratio of CaO to SiO_2_ (C/S ratio) in its structure, which can be used to reflect the changes in the chemical composition of the C-S-H colloids in the ITZ and cement pastes. The C/S ratio is also an important indicator to distinguish the rich phases of hydration products [25,26]. According to some studies [27,28], a lower C/S ratio of the ITZ generally indicates a higher content of C-S-H colloids and a lower content of CH in the cement matrix. As for the vicinity of the aggregate, the C/S ratio increases due to the presence of large CH crystals [29]. In general, C/S ratios between 0.8 and 2.5 can be considered as C-S-H-colloid-rich hydrates, C/S ratios higher than 4 are considered to be rich in monosulfates (AFm), and C/S ratios higher than 10 are considered to be rich in calcium hydroxide (CH) [25]. When the C/S ratio is decreased below 1.0, the BET (Brunauer–Emmett–Teller) surface area of the C-S-H substantially increases, and these properties have been shown to affect the physicochemical behavior of C-S-H [30]. In the experimental-group specimens, the C/S ratio was 0.87, which indicates that it was rich in the hydrates of C-S-H colloids.

#### 3.3.2. FESEM Observation and EDS Analysis after 28 Days of Self-Healing

**Figure 13:**
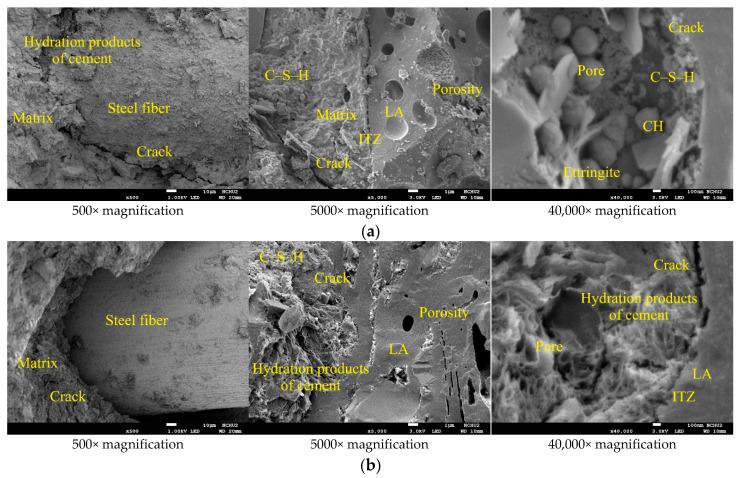
SEM micrographs of the concrete specimens after self-healing for 28 days: (**a**) control group; (**b**) experimental group.

**Figure 14:**
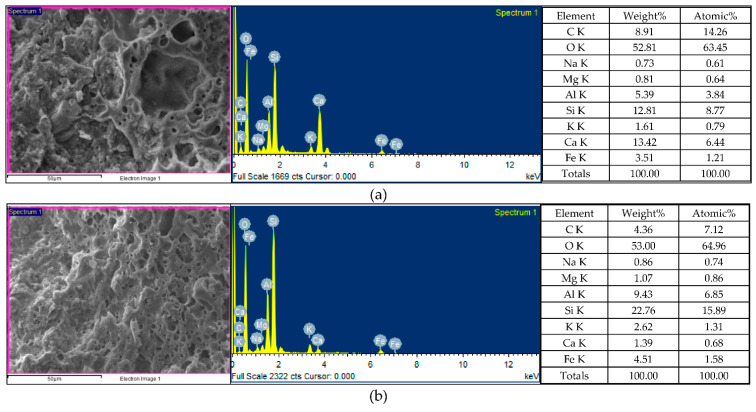
EDS analysis of the concrete specimens after self-healing for 28 days: (**a**) control group; (**b**) experimental group.

After 28 days of self-healing, the FESEM micrographs of the concrete specimens revealed information on their morphological changes and the formation of rehydration products, as shown in Figure 13. Compared with the specimens cured for 14 days, the micropores of the concrete specimens were reduced and the microstructures appeared denser due to the continuous rehydration of the uncemented cement. In other words, the longer the self-healing period of concrete, the more substantial its strength recovery. It can observe that the interface between the matrix and steel fiber in Figure 13. The higher magnification of Figure 13 revealed the ITZ between the matrix and lightweight aggregate, as well as the cement-hydration products. The elongated hexagonal needles were ettringite, the hexagonal flakes were calcium hydroxide, and the network flocs were C-S-H colloids (the black parts were the voids of the filler). In addition, many fine pores were observed inside the light aggregates. After self-healing for 28 days, the EDS spectra of the concrete sample are shown in Figure 14.

According to the EDS analysis of the control-group specimens (Figure 14a), they contained the following nine elements—C, O, Na, Mg, Al, Si, K, Ca, and Fe—among which the main elements (the percentage by weight) were O (52.81%), Ca (13.42%), Si (12.81%), and C (8.91%). According to the EDS analysis of the experimental-group specimens (Figure 14b), they contained the following nine elements: C, O, Na, Mg, Al, Si, K, Ca, and Fe, among which the main elements (the percentage by weight) were O (53.0%), Si (22.76%), Al (9.43%), and Fe (4.51%). It was found that a higher percentage of elemental silicon in the experimental-group specimens. In general, the C/S ratio is an important parameter for the microstructure and mechanical properties of C-S-H. The C/S ratio of the concrete specimens changed with the increase in the curing age and the degree of the hydration. In the control group, the C/S ratio was 0.73. In the experimental group, the C/S ratio was 0.04.

#### 3.3.3. XRD Analysis

**Figure 15:**
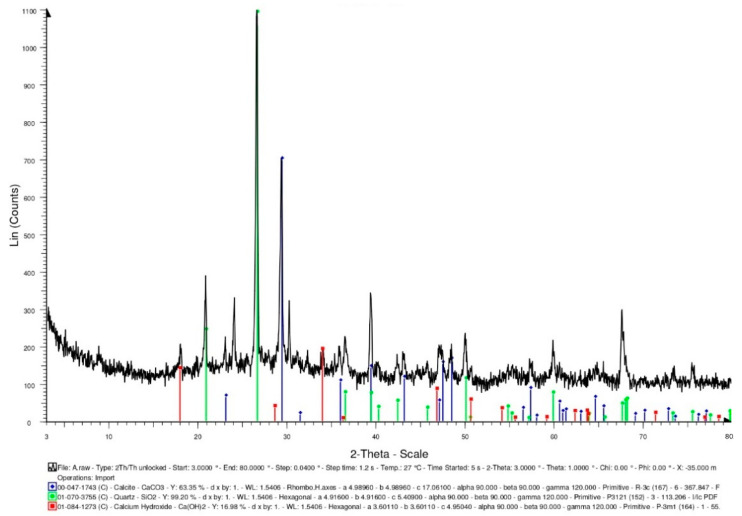
XRD-analysis results of the control group specimen.

**Figure 16:**
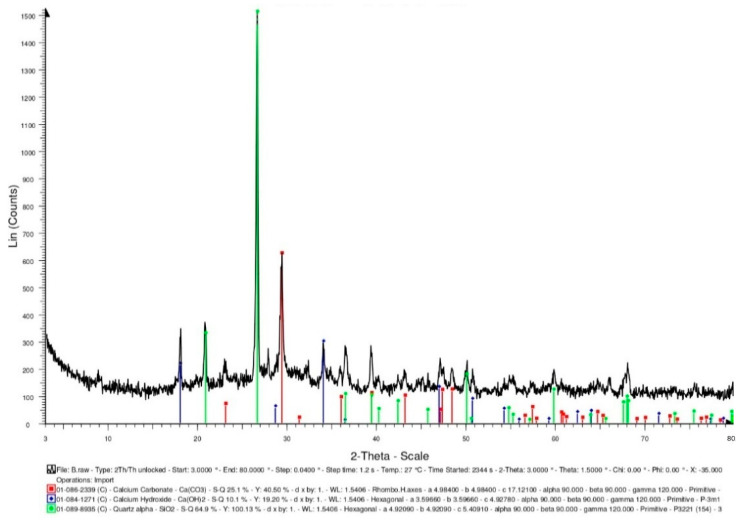
XRD-analysis results of the experimental group specimen.

XRD-analysis results can provide information about the phases and compounds that are present in specimens. Therefore, XRD analysis was performed on the control group and experimental group specimens to determine the mineral compositions. In the control group specimen, it was able to confirm the presence of calcite crystals (observed at 2-theta of 29.4°), and its X-ray-reflection-energy intensity was 708 (unit: counts), as shown in Figure 15. In addition, the XRD spectra revealed the presence of quartz with hydrated calcium silicate and calcium hydroxide. In the experimental-group specimen, it was also observed a prominent crystalline peak of CaCO_3_ at 2-theta of 29.4°, and its X-ray-reflection-energy intensity was 629 (unit: counts), as shown in Figure 16. Moreover, the XRD spectra revealed the presence of quartz alpha and calcium hydroxide. According to the above results, due to the further hydration of the unhydrated cement particles and the precipitation of calcium carbonate crystals at the smaller-width cracks, a small amount of healing product was formed, which allowed the concrete to spontaneously heal.

### 3.4. Results of Compressive Strength Test

#### 3.4.1. Compressive Strength of Concrete upon Initial Curing

Table 7 shows the 28-day compressive strength test results of each group of concrete at room temperature. The 28-day compressive strengths of the two groups of concrete were approximately equal. The compressive strengths of the control group and the experimental group were 37.08 and 34.78 MPa, respectively. The 28-day elastic modulus of each group of concrete at room temperature is shown in Table 7. The elastic moduli of the control group and the experimental group were 25.94 and 28.57 GPa, respectively.

**Figure 17:**
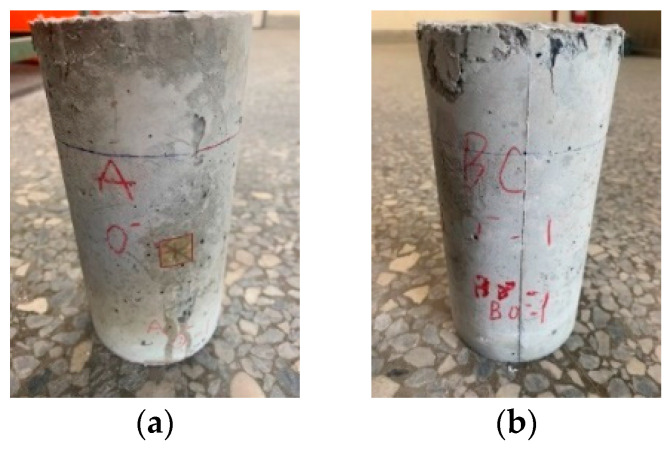
Destruction appearance of the concrete after compressive test at room temperature: (**a**) control group specimens; (**b**) experimental group specimen.

During the compressive test, the two groups of concrete specimens did not burst when subjected to the ultimate load due to the hooking or bridging effect of the steel fibers. Therefore, the specimens in the control group and experimental group still maintained the original cylindrical shape after being destroyed, as shown in Figure 17.

Concrete curing is a process that maintains proper humidity and temperature conditions to ensure continuous cement hydration [1]. The properties of concrete will improve with age if the environment is suitable for hydration to continue [2]. According to this, the concrete specimens in each group that were damaged by compression at room temperature were cured in the laboratory curing tank. The results of the second compressive strength test after self-healing for 28 days are shown in Table 8. The secondary compressive strengths of the control group and the experimental group were 27 and 28.57 MPa, respectively. Furthermore, the compressive strength of the specimens upon initial curing were used as the benchmark, and the residual compressive strengths after self-healing were divided by the original compressive strengths at room temperature to calculate the relative-compressive-strength ratios. It can be clearly seen from Table 9 that the relative-compressive-strength ratios of the control group and the experimental group after self-healing for 28 days were 0.73 and 0.82, respectively. Compared with the specimens in the control group, the relative-compressive-strength ratios of the specimens in the experimental group increased by 12.8%, which indicates that the strength recoveries of the specimens treated with MICP were better. The relative-elastic-modulus ratios for the specimens with the 28-day self-healing for compressive failure were also calculated, shown in Table 10. The relative-elastic-modulus ratios of the control group and experimental group were 0.75 and 0.62, respectively. Compared with the specimens in the control group, the relative-elastic-modulus ratios of the specimens in the experimental group decreased by 17.4%.

#### 3.4.2. Compressive Strength of Concrete after Exposure to High Temperature

At the age of 28 days, the specimens of the two groups of concrete were subjected to a high-temperature test. After the exposure to high temperatures, the deterioration of the concrete groups, such as color change, surface delamination, cracking, etc. Possible causes of these degradations included thermal gradients, the evaporation of free water, and chemical changes in concrete [23,31,32]. In terms of the chemical changes, weakened bonds and increased voids lead to reduced mechanical properties of concrete due to the evaporation of crystal water and the decarburization of carbonates [33]. Table 11 shows the results of the compressive-strength test for the control group and experimental group after the high-temperature test. The test results of the control group and experimental group were quite similar. The compressive strengths of the control group and experimental group were 35.78 MPa and 35.15 MPa, respectively. As for the elastic modulus after exposure to high temperature, there was no significant difference between the test results of the control group and the experimental group. The elastic moduli of the control group and experimental group were 19.72 GPa and 20.38 GPa, respectively.

After being exposed to 500 °C and cooled to room temperature, the residual compressive strength test was carried out. Based on the compressive strength of the specimen at room temperature, the residual compressive strengths after the exposure to high temperature were divided by the compressive strengths at room temperature to calculate the relative-compressive-strength ratios. The results are shown in Table 12. The relative-compressive-strength ratios of the control group and the experimental group were 0.97 and 1.01, respectively. Overall, the compressive strengths of the control group and the experimental group had no obvious decline after exposure to the high temperature of 500 °C. As for the elastic modulus, the results of the relative-elastic-modulus ratios are shown in Table 13. The relative-elastic-modulus ratios of the control group and the experimental group were 0.76 and 0.71, respectively.

#### 3.4.3. Compressive Strength of Self-Healing Concrete after Exposure to High Temperature

After the high-temperature test, the test results of the compressive strength of the self-healing concrete are shown in Table 14. The compressive strengths of the control group after self-healing for 14 and 28 days were 35.75 MPa and 34.91 MPa, respectively. The compressive strengths of the experimental group after self-healing for 14 and 28 days were 33.34 MPa and 33.06 MPa, respectively. There was a clear difference in the test results for the elastic modulus after self-healing between the control group and experimental group. The elastic moduli of the control group after self-healing for 14 and 28 days were 18.02 GPa and 21.32 GPa, respectively. The elastic moduli of the experimental group after self-healing for 14 and 28 days were 21.24 GPa and 25.28 GPa, respectively. 

Taking the compressive strength after exposure to high temperature as the benchmark, the relative-compressive-strength ratio of the specimens subjected to different self-healing ages after high temperature is calculated, as shown in Table 15. The relative-compressive-strength ratios of the control group after self-healing for 14 and 28 days were 1.00 and 0.98, respectively. The relative-compressive-strength ratios of the experimental group after self-healing for 14 and 28 days were 0.95 and 0.94, respectively. Compared with the control group, the relative-compressive-strength ratio of the experimental group after self-healing for 28 days decreased by 3.6%. It is possible that the high temperature caused the loss of bacteria in the experimental group, resulting in insignificant mineralization. 

Furthermore, for the specimens subjected to different curing ages after the exposure to high temperature, the relative-elastic-modulus ratios were calculated, as shown in Table 16. The relative-elastic-modulus ratios of the control group after self-healing for 14 and 28 days were 0.91 and 1.08, respectively. The relative-elastic-modulus ratios of the experimental group after self-healing for 14 and 28 days were 1.04 and 1.24, respectively. Compared with the control group, the relative-elastic-modulus ratio of the experimental group after self-healing for 28 days increased by 14.7%. According to these results, the elastic modulus values of the specimens in the experimental group increased after different self-healing ages. This may be related to the mineralization of bacteria.

#### 3.4.4. Influences of Different Test Parameters on Compressive Strength

**Figure 18:**
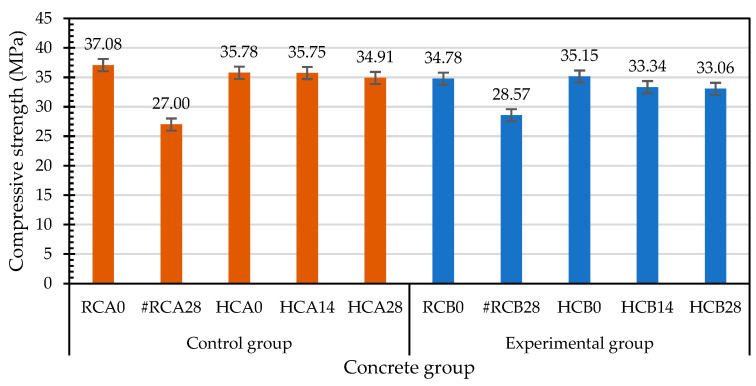
Effects of different test parameters on the compressive strengths of concrete.

The overall performance of concrete is the result of the combination of its mechanical properties and durability, which is closely related to the way the concrete is cured [34,35]. The curing efficiency of concrete is related to the humidity and temperature of the environment, as well as the exposure time of the concrete in the early stage of composite hydration [36]. The compressive strengths of each group of concrete were different under different test parameters, as shown in Figure 18. The results of the second compressive-strength test after self-healing for 28 days for each group of compressive-failure specimens were substantially lower. For each group of concrete exposed to high temperature, the residual compressive strength after self-healing was greater than 33 MPa, which indicates that curing in water has a certain effect. In fact, concrete exposed to high temperatures can recover a large proportion of the original strength of fire-damaged concrete without repair [24,37,38,39,40]. The specimens of the control group and experimental group were immersed in the curing tank of the laboratory for self-healing, and the calcium oxide and unhydrated cement particles in the specimens could absorb the water from the surrounding medium for rehydration. The residual compressive strengths of these two groups of specimens could maintain a certain strength due to the filling of the micropores by the rehydration product [38]. Pan et al. [24] proposed that high temperatures open capillaries that were initially blocked by hydration products. During the self-healing process, these capillaries were refilled with hydration products. These hydration products were smaller in size than the original hydration products, resulting in a finer pore structure that helped the concrete regain its strength and durability.

**Figure 19:**
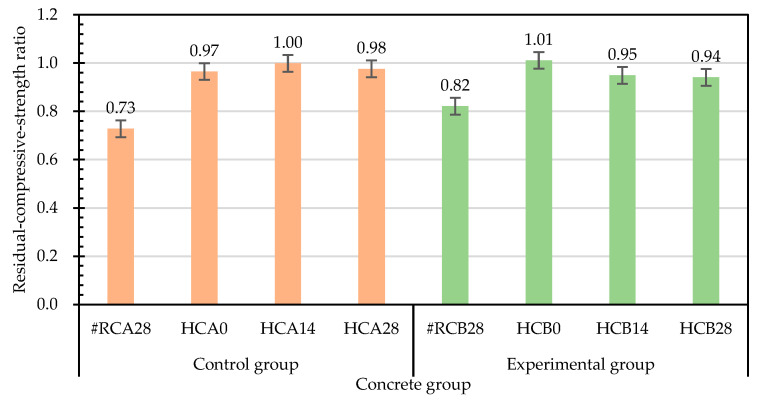
Effects of different test parameters on the relative-compressive-strength ratios of concrete.

The relative-compressive-strength ratios of each group of concrete cured before and after the exposure to 500 °C are shown in Figure 19. The relative-compressive-strength ratios of the compressive-failure specimens of each group after self-healing for 28 days were significantly lower. As for the specimens exposed to 500 °C, the relative-compressive-strength ratios of the control group and experimental group at different self-healing ages ranged from 0.97 to 1.0 and from 0.94 to 1.01, respectively. Specimens from the control and experimental groups were immersed in a laboratory curing tank. This type of curing avoids the loss of the internal mixing water and optimizes the cement hydration to form a hydrated-calcium-silicate network to provide the concrete strength. This is consistent with the findings of Cremonez et al. [41].

**Figure 20:**
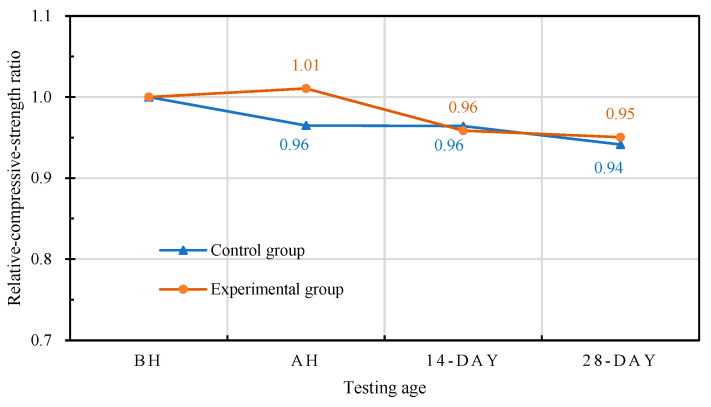
Effects of different self-healing ages on the relative-compressive-strength ratios of concrete.

Taking the 28-day-old compressive strength (before high temperature (BH)) at room temperature as the benchmark for comparison, the relative-compressive-strength ratios of each group of concrete at different self-healing ages are shown in Figure 20. The residual compressive strengths (after high temperature (AH)) of the specimens in the experimental group were higher than the compressive strengths at room temperature. However, with the increase in the self-healing age, the relative-compressive-strength ratios decreased. The residual compressive strength of the control group after the exposure to high temperature was lower than the compressive strength at normal temperature. With the increase in the self-healing age, the relative-compressive-strength ratios slowly decreased. During curing, the initial decrease in the strength at 14 days of self-healing may have been due to the relative expansion of the wetted outer layer of the concrete, and the subsequent recovery at 28 days of self-healing was due to the regeneration of the C-S-H bonds after rehydration [42]. Each group of concrete specimens showed a certain degree of compressive-strength recovery after self-healing. According to this result, the compressive-strength recovery depends on the self-healing of the concrete. When the fire-damaged concrete was in contact with water after cooling, the micropores could be filled due to the regeneration of the C-S-H and carbonate phases, and the cracks could be healed and the strength of the concrete recovered, which is consistent with the results of Poon et al. [24]. However, water recuring is a controversial approach, as some researchers have found that water recuring is detrimental to concrete [24,43]. For example, CaO in concrete rehydrates it, causing it to disintegrate and further deteriorate. Lin et al. [38] pointed out that Ca(OH)_2_ was produced at the end of rehydration, and its volume was 44% higher than that of CaO. This expansion led to an increase in the size and number of existing cracks in the concrete, which further reduced its mechanical properties upon cooling [25].

### 3.5. Results of Flexural Strength Test

#### 3.5.1. Flexural Strength of Concrete at upon Initial Curing

**Figure 21:**
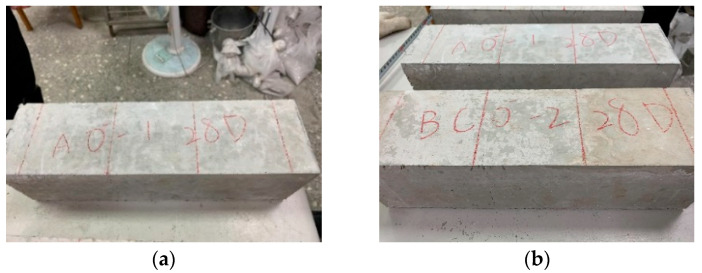
Destruction appearance of the concrete specimens after flexural test at room temperature: (**a**) control group; (**b**) experimental group.

The results of the 28-day flexural test of each group of concrete at room temperature are shown in Table 17. The flexural strengths of the control group and experimental group were 4.50 MPa and 4.36 MPa, respectively. In addition, the deflections at the midspans of the control group and experimental group were 0.419 mm and 0.428 mm, respectively. Figure 21 shows the failure of each group of specimens after the flexural test. The specimens of the control group and experimental group could still maintain their original appearance and exhibited high toughness after the failure. During the test, the load-carrying capacity continued to increase after the formation of the first crack, and all the specimens exhibited deflection hardening properties. When the specimen reached the ultimate flexural load, it could still maintain a certain toughness and strength, which avoided the formation of brittle failure. The reason is that the short steel fibers absorbed the tensile stress to prevent the expansion and coherence of the microcracks, thereby preventing the formation of macrocracks. Under the continuous action of a load, cracks or microcracks will form in concrete. Adding a large number of short steel fibers (0.75% by volume) can form bridging and hook effects with the concrete, avoiding the formation of large local strains.

On the other hand, the specimens damaged by the flexural load at room temperature in each group were cured in the laboratory curing tank. The results of the second flexural strength test after self-healing for 28 days are shown in Table 18. The flexural strengths of the control group and the experimental group were 1.96 MPa and 1.53 MPa, respectively. The deflections at the midspan of the control group and the experimental group were 0.864 mm and 0.830 mm, respectively. In addition, based on the flexural strength of the specimen at room temperature, the residual flexural strength after self-healing for 28 days was divided by the flexural strength at room temperature to calculate the relative-flexural-strength ratio, as shown in Table 19. The relative-flexural-strength ratios of the specimens in the control group and the experimental group were 0.44 and 0.35, respectively.

#### 3.5.2. Flexural Strength of Concrete after Exposure to High Temperature

Table 20 shows the flexural test results of the specimens in the control group and the experimental group after exposure to high temperature. The flexural strengths of the control group and the experimental group were 2.86 MPa and 2.61 MPa, respectively. Based on the flexural strengths of the specimens at room temperature, the relative-flexural-strength ratios were calculated by dividing the uncured residual flexural strengths after exposure to high temperature by the flexural strengths at room temperature, as shown in Table 21. The residual flexural strength of the control group was 63.6% of its room-temperature flexural strength, whereas the experimental-group flexural strength was 59.9% of its room-temperature flexural strength. Overall, the flexural strengths of the control group and experimental group substantially decreased after the exposure to a high temperature of 500 °C.

#### 3.5.3. Flexural Strength of Self-Healing Concrete after Exposure to High Temperature

After the high temperature test, the flexural strength of the two groups of concrete specimens after self-healing are shown in Table 22. The flexural strengths of the control group and experimental group were 3.28 MPa and 2.90 MPa, respectively. The midspan deflections of the control group and experimental group were 0.321 mm and 0.313 mm, respectively. In addition, for the self-healing specimens for 28 days after exposure to high temperature, the relative-flexural-strength ratios based on their flexural strengths after the exposure to high temperature were calculated. The relative-flexural-strength ratios of the control group and experimental group were 1.15 and 1.11, respectively, shown in Table 23. As previously speculated, it was the high temperature that caused the loss of bacteria in the experimental group, resulting in insignificant mineralization.

The results of the second flexural strength test after self-healing for 28 days are shown in Table 24. The flexural strengths of the control group and experimental group were 2.02 MPa and 1.27 MPa, respectively. The net midspan deflections of the control group and experimental group were 1.141 mm and 0.719 mm, respectively. Furthermore, the relative-flexural-strength ratios were calculated for the specimens that had been cured for 28 days after exposure to 500 °C. The relative-flexural-strength ratios of the control group and the experimental group were 0.71 and 0.49, respectively, shown in Table 25.

#### 3.5.4. Influences of Different Test Parameters on Flexural Strength

**Figure 22:**
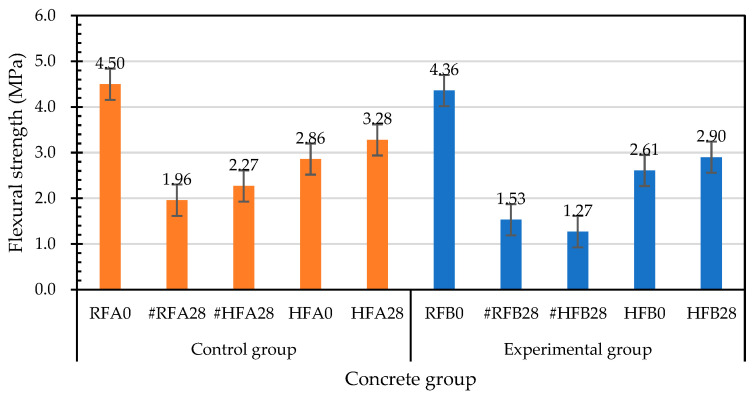
Effects of different test parameters on the flexural strengths of concrete.

Several studies have pointed out that the mechanical properties degradation of concrete exposed to high temperature was caused by a series of physicochemical changes, especially the dehydration and decomposition of hydrated cement paste during heating [25,38]. According to the above analysis, under different test parameters, there were obvious differences in the flexural strengths of each group of concrete. At room temperature, the secondary flexural-strength-test results of each group of flexural-failure specimens after self-healing for 28 days were low (Figure 22). According to the results of the secondary flexural-strength test after self-healing for 28 days, the flexural strengths of the flexural-failure specimens of both the control group and experimental group after exposure to a high temperature substantially decreased. For each group of concrete exposed to high temperatures, the residual flexural strength after self-healing was greater than 2.61 MPa, which indicates that the curing had a certain effect. After the exposure to high temperatures, the recoveries of the flexural strengths of the specimens during curing were due to the rehydration of the decomposed hydration products, and to the further hydration of the initially unhydrated cement particles, which is consistent with the results of Khoury [37].

**Figure 23:**
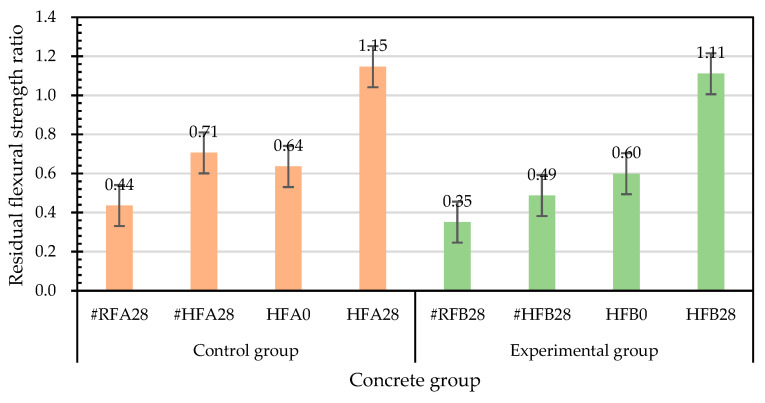
Effects of different test parameters on the relative-flexural-strength ratios of concrete.

The relative-flexural-strength ratios of each group of concrete cured before and after exposure to high temperature are shown in Figure 23. The relative-flexural-strengths of the flexural-failure specimens of each group after curing for 28 days under normal-temperature conditions were significantly lower. Similarly, the relative-flexural-strength ratios of the flexural-failure specimens of each group after self-healing for 28 days were also significantly lower. The relative-flexural-strength ratios of the cured specimens after exposure to high temperature were 1.15 for the control group, and 1.11 for the experimental group. According to this result, the flexural strength of each group of flexural specimens cured for 28 days after exposure to high temperatures improved.

**Figure 24:**
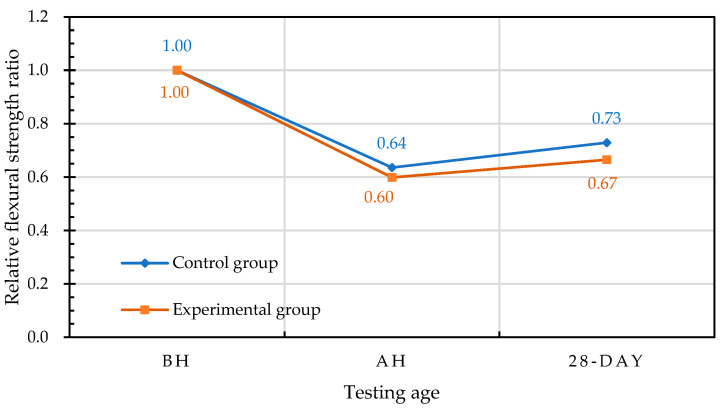
Effects of different curing ages on the relative-flexural-strength ratios of concrete.

Taking the 28-day-old flexural strength (before high temperature (BH)) at room temperature as the benchmark for comparison, the relative-flexural-strength ratios of each group of concrete at different self-healing ages are shown in Figure 24. The residual flexural strength (after high temperature (AH)) of the specimen in the experimental group after the exposure to high temperature was lower than the flexural strength at room temperature, and its relative-flexural-strength ratio was 0.60, whereas the relative-flexural-strength of the specimen after self-healing for 28 days was 0.67. The residual flexural strength of the control group after exposure to 500 °C was lower than the flexural strength at room temperature, and its relative-flexural-strength ratio was 0.64, whereas the relative-flexural-strength ratio of the self-healing for 28 days was 0.73.

#### 3.5.5. Crack Healing Analysis

In order to understand the healing of the cracks in the concrete flexural specimens after curing, a specific crack was selected and observed with a crack-width-measuring instrument. For each group of concrete specimens that were flexurally cracked after exposure to high temperature, photographs of the initial cracks on the surface were first taken. Then, the specimens were placed in a water tank for self-healing. When the self-healing age was reached, the specimens were taken out and the crack width was measured. The crack-width-measuring instrument can be used to enlarge the crack image and accurately measure the crack-width value, thereby allowing for a clear observation of the crack healing of the specimens.

**Figure 25:**
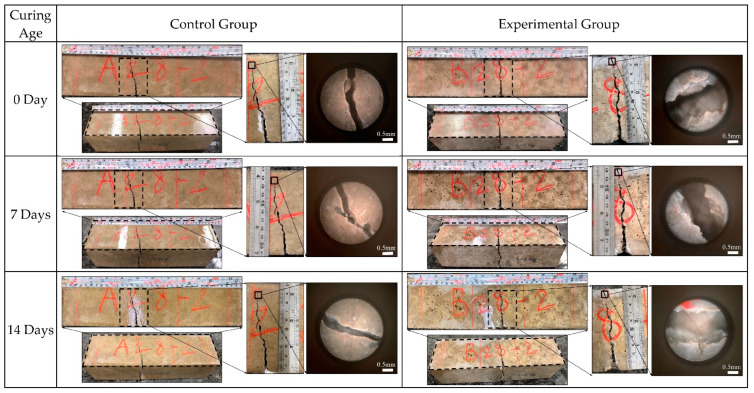
Microscopic images of crack healing of the concrete specimens at different self-healing ages.

Figure 25 shows the microscopic images of the surface cracks of each group of specimens at different healing times. According to the measurement results, the initial surface-crack widths of each group of concrete specimens were much higher than the maximum limit specified by the ACI Building Code (0.10 mm for wet conditions, and 0.44 mm for dry conditions). The crack widths of different concrete specimens decreased gradually with the self-healing age. The healing precipitation at the cracks was observed. After the specimens of the control group and the experimental group were cured in the water tank of the curing room for 14 days, their crack widths gradually healed. This is because secondary water curing promotes the further hydration of unhydrated cement particles and the precipitation of calcium carbonate crystals at smaller-width cracks, the rehydration products of which can fill the cracks and pores [44]. According to this result, the cracks between the cement matrix and aggregates can be repaired by secondary water curing in the water tank [45]. The crack widths of the specimens in the experimental group are not substantially reduced. According to this result, the mineralization of the bacteria with the light aggregates as carriers was not substantial under the curing environment of the water tank without a nutrient source. Another possible reason is that the effect of the high temperature on the survival of the bacteria in the experimental group resulted in slow mineralization.

### 3.6. Results of Water Penetration Test

#### 3.6.1. Water Absorption of Concrete upon Initial Curing

Essentially, the useful life of concrete is usually determined by its durability properties, which are related to its water absorption. In view of this, the water absorption of each group of concrete specimens was measured. Table 26 shows the results of the 28-day water-penetration test of the concrete at room temperature of the control group and experimental group. The water absorption rates of the control group and experimental group were 3.3% and 3.2%, respectively. According to these results, there was no difference in the permeability of the concrete between the control group and experimental group at room temperature.

#### 3.6.2. Water Absorption of Concrete after Exposure to High Temperature

**Figure 26:**
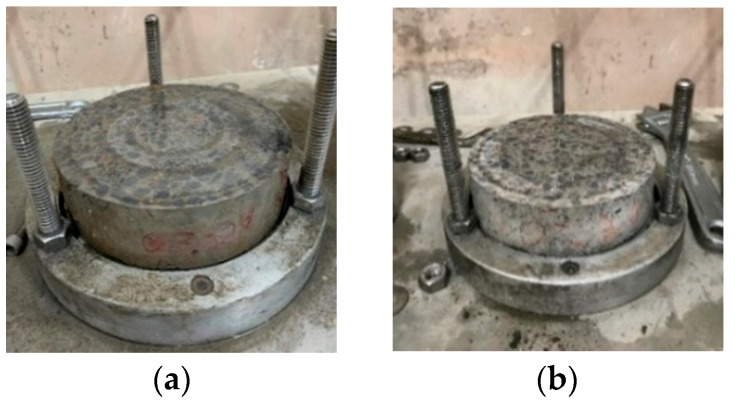
Appearance of the surface of the specimens exposed to high temperature after the water-penetration test: (**a**) control group; (**b**) experimental group.

After exposure to high temperature, the water penetration test results of the control group and the experimental group are shown in Table 27. The water-absorption rates of the control group and experimental group were 10.3% and 10.9%, respectively. From this point of view, the water absorption of the control group and experimental group after exposure to high temperature substantially increased, which was caused by the formation of microcracks on the surface of the specimens, internal cracks, and the coarsening of the pore structure caused by the high temperature. The appearance of the water adsorbed on the surface of each group of specimens after the water penetration test is shown in Figure 26. The control group adsorbed less water, whereas the experimental group adsorbed more water.

#### 3.6.3. Water Absorption of Self-Healing Concrete after Exposure to High Temperature

After the high temperature test, the water permeability test results of the control group and the experimental group after curing for 28 days are shown in Table 28. The water-absorption rates of the control group and experimental group were 6.7% and 5.6%, respectively. This is because the unhydrated silicate was hydrated into calcite, self-healing occurred. Compared with the results of the compressive and flexural tests, the results of the water penetration test of the experimental group were better than those of the control group. This is because the specimens of the water penetration test were taken from the middle part of the cylindrical specimens after high temperature, and their bacterial survival rate was relatively high. Therefore, the mineralization of bacteria was more significant, which made the experimental group more compact, thereby reducing the water absorption rate.

#### 3.6.4. Influences of Different Test Parameters on Water Absorption

**Figure 27:**
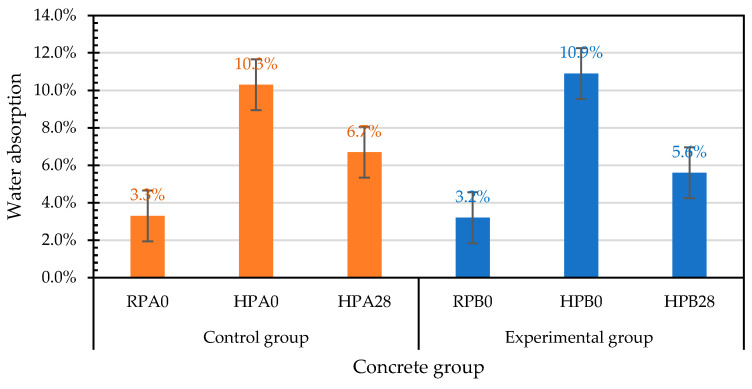
Effects of different test parameters on water absorption of concrete.

According to the above analysis, there were obvious differences in the penetration-test results of each group of concrete under different test parameters, as shown in Figure 27. There was no significant difference in the water-absorption rate of each group of concrete under normal-temperature conditions (control group: 3.3%; experimental group: 3.2%). After the exposure to high temperature, the water-absorption rate of the concrete substantially increased. The water-absorption rates of the control group and experimental group were 10.3% and 10.9%, respectively. After the exposure to high temperature and self-healing for 28 days, the water-absorption rate of each group of concrete was greatly reduced, and the water-absorption rates of the control group and experimental group were 6.7% and 5.6%. The reason for this is that the rehydration-reaction product filled the capillaries and internal cracks, which reduced the water permeability of each group of concrete after curing. For each group of concrete exposed to high temperature, the water permeability after curing was greatly reduced, which meant that the curing had a certain effect.

## 4. Conclusions

According to the above test results and analyses, the following conclusions were obtained.

- After being exposed to a high temperature of 500 °C, the relative-compressive-strengths of the control group and experimental group were 97% and 101% of the room-temperature compressive strengths, respectively. The relative-compressive-strength ratios of the control group and experimental group at different self-healing ages ranged from 0.97 to 1.0 and from 0.94 to 1.01, respectively.
- After exposure to high temperature, the relative-flexural-strengths of the control group and experimental group were 64% and 60% of the room-temperature flexural strengths, respectively. The relative-flexural-strength ratios of the control group and experimental group at different self-healing ages ranged from 0.64 to 1.15 and from 0.60 to 1.11, respectively.
- After self-healing for 28 days, the water-absorption rates of the control group and experimental group were 6.7% and 5.6%, respectively. This is because the specimens of the water penetration test were taken from the middle part of the cylindrical specimens after high temperature, and their bacterial survival rate was relatively high. Therefore, the mineralization of bacteria was more significant, thereby reducing the water absorption rate of the experimental group.
- The strength recovery of the concrete after exposure to high temperature was closely related to the curing environment. When the fire-damaged concrete was in contact with water after cooling, the micropores could be filled due to the regeneration of the C-S-H and carbonate phases, which could heal the cracks and restore the strength of the concrete.
- The EDS and XRD analyses confirmed that the precipitate formed at the crack was calcium carbonate. Therefore, each group of concrete specimens showed a certain degree of strength recovery after self-healing.
- Overall, after exposure to a high temperature of 500 °C, there was no significant difference in the mechanical test results of the control group and the experimental group after self-healing in water. This is because the high temperature caused the loss of bacteria in the experimental group, resulting in insignificant mineralization. Furthermore, in the absence of an adequate source of nutrients, the mineralization of bacteria was slow. Subsequent research could focus on how to provide a source of nutrients to ensure the efficient functioning of biomineralization.

## Figures and Tables

**Table 1:** Basic physical and mechanical properties of fibers.

Fiber Type	Length (mm)	Diameter (mm)	Density (g/cm^3^)	Elastic Modulus (GPa)	Tensile Strength (MPa)	Melting Point (°C)
Steel fibers	13	0.2	7.8	200	2000	-
Polypropylene fibers	12	0.05	0.9	-	300	165

**Table 2:** Mix proportions of the concretes.

Concrete Group	W/B	W (kg/m^3^)	C (kg/m^3^)	LA (kg/m^3^)	FA (kg/m^3^)	SF (kg/m^3^)	PP (kg/m^3^)	SP (kg/m^3^)
Control group	0.467	202.7	431.2	667.4	548.4	58.5	0.819	0.517
Experimental group	0.467	202.7	431.2	667.4	548.4	58.5	0.819	0.517

Notes: W/B: water–binder ratio; W: water; C: cement; LA: lightweight aggregate; FA: fine aggregate; SF: steel fiber; PP: polypropylene fiber; SP: superplasticizer.

**Table 3:** Compression test items and test sequence.

Test Item	Designation	Test Sequence
Compressive strength test upon initial curing	RCA0	Loading
	RCB0	
Compression strength test after self-healing of compressive failure specimen	#RCA28	Loading → Self-Healing → Reloading
	#RCB28	
Compressive strength test after high temperature	HCA0	Heating → Loading
	HCBC0	
Compressive strength test of self-healing concrete after high temperature	HCA14	Heating → Self-Healing → Loading
	HCA28	
	HCB14	
	HCB28	

**Table 4:** Flexural test items and test sequence.

Test Item	Designation	Test Sequence
Flexural strength test upon initial curing	RFA0	Loading
	RFB0	
Flexural strength test after self-healing of flexural failure specimen	#RFA28	Loading → Self-Healing → Reloading
	#RFB28	
Flexural strength test after high temperature	HFA0	Heating → Loading
	HFB0	
Flexural strength test of self-healing concrete after high temperature	HFA28	Heating → Self-Healing → Loading
	HFB28	

**Table 5:** Water penetration test items and test sequence.

Test Item	Designation	Test Sequence
Water penetration test upon initial curing	RPA0	Penetrating
	RPB0	
Water penetration test after high temperature	HPA0	Heating → Penetrating
	HPB0	
Water penetration test of self-healing concrete after high temperature	HPA28	Heating → Self-Healing → Penetrating
	HPB28	

**Table 6:** Fresh properties of the concretes.

Concrete Group	Slump (cm)	Slump Flow (cm)	Unit Weight (kg/m^3^)
Control group	6	20	1909.5
Experimental group	6	20	1909.5

**Table 7:** Test results of compressive strength and elastic modulus of the concrete at room temperature.

Concrete Group	Designation	Compressive Strength (MPa)	Elastic Modulus (GPa)
Control group	RCA0	37.08	25.94
Experimental group	RCB0	34.78	28.57

**Table 8:** Result of secondary compressive test of the compressive failure specimen after self-healing.

Concrete Group	Designation	Compressive Strength (MPa)	Elastic Modulus (GPa)
Control group	#RCA28	27.00	19.37
Experimental group	#RCB28	28.57	17.63

**Table 9:** Relative-compressive-strength ratio of the compressive failure specimen after self-healing.

Concrete Group	Compressive Strength at Room Temp. (MPa)	Residual Compressive Strength (MPa)	Relative-Compressive-Strength Ratio
Control group	37.08	27.00	0.73
Experimental group	34.78	28.57	0.82

**Table 10:** Relative-elastic-modulus ratio of the compressive failure specimen after self-healing.

Concrete Group	Elastic Modulus at Room Temp. (GPa)	Residual Elastic Modulus (GPa)	Relative-Elastic-Modulus Ratio
Control group	25.94	19.37	0.75
Experimental group	28.57	17.63	0.62

**Table 11:** Test results of compressive strength and elastic modulus of the concrete after exposure to high temperature.

Concrete Group	Designation	Compressive Strength (MPa)	Elastic Modulus (GPa)
Control group	HCA0	35.78	19.72
Experimental group	HCB0	35.15	20.38

**Table 12:** Relative-compressive-strength ratio of the concrete after exposure to high temperatures.

Concrete Group	Compressive Strength at Room Temp. (MPa)	Residual Compressive Strength (MPa)	Relative-Compressive-Strength Ratio
Control group	37.08	35.78	0.97
Experimental group	34.78	35.15	1.01

**Table 13:** Relative-elastic-modulus ratio of the concrete after exposure to high temperatures.

Concrete Group	Elastic Modulus at Room Temp. (GPa)	Residual Elastic Modulus (GPa)	Relative-Elastic-Modulus Ratio
Control group	25.94	19.72	0.76
Experimental group	28.57	20.38	0.71

**Table 14:** Test results of compressive strength and elastic modulus of the self-healing concrete after exposure to high temperature.

Concrete Group	Designation	Compressive Strength (MPa)	Elastic Modulus (GPa)
Control group (curing: 14 days)	HCA14	35.75	18.02
Experimental group (curing: 14 days)	HCB14	33.34	21.24
Control group (curing: 28 days)	HCA28	34.91	21.32
Experimental group (curing: 28 days)	HCB28	33.06	25.28

**Table 15:** Relative-compressive-strength ratios of the self-healing concrete after exposure to high temperature.

Concrete Group	Compressive Strength after High Temp. (MPa)	Residual Compressive Strength (MPa)	Relative-Compressive-Strength Ratio
Control group (curing: 14 days)	35.78	35.75	1.00
Experimental group (curing: 14 days)	35.15	33.34	0.95
Control group (curing: 28 days)	35.78	34.91	0.98
Experimental group (curing: 28 days)	35.15	33.06	0.94

**Table 16:** Relative-elastic-modulus ratios of the self-healing concrete after exposure to high temperature.

Concrete Group	Elastic Modulus after High Temp. (MPa)	Residual Elastic Modulus (MPa)	Relative-Elastic-Modulus Ratio
Control group (curing: 14 days)	19.72	18.02	0.91
Experimental group (curing: 14 days)	20.38	21.24	1.04
Control group (curing: 28 days)	19.72	21.32	1.08
Experimental group (curing: 28 days)	20.38	25.28	1.24

**Table 17:** Results of flexural test of the concrete at room temperature.

Concrete Group	Designation	Flexural Strength (MPa)	Deflection at Midspan (mm)
Control group	RFA0	4.50	0.419
Experimental group	RFB0	4.36	0.428

**Table 18:** Results of secondary flexural test of the flexural failure specimens after self-healing.

Concrete Group	Designation	Flexural Strength (MPa)	Deflection at Midspan (mm)
Control group	#RFA28	1.96	0.864
Experimental group	#RFB28	1.53	0.830

**Table 19:** Relative-flexural-strength ratios of the flexural failure specimens after self-healing.

Concrete Group	Flexural Strength at Room Temp. (MPa)	Residual Flexural Strength (MPa)	Relative-Flexural-Strength Ratio
Control group	4.50	1.96	0.44
Experimental group	4.36	1.53	0.35

**Table 20:** Flexural strength test results of the concrete after exposure to high temperature.

Concrete Group	Designation	Flexural Strength (MPa)	Deflection at Midspan (mm)
Control group	HFA0	2.86	0.334
Experimental group	HFB0	2.61	0.357

**Table 21:** Relative-flexural-strength ratio of the concrete after exposure to high temperature.

Concrete Group	Flexural Strength at Room Temp. (MPa)	Residual Flexural Strength (MPa)	Relative-Flexural-Strength Ratio
Control group	4.50	2.86	0.64
Experimental group	4.36	2.61	0.60

**Table 22:** Test results of the flexural strength of the self-healing concrete after exposure to high temperature.

Concrete Group	Designation	Flexural Strength (MPa)	Deflection at Midspan (mm)
Control group	HFA28	3.28	0.321
Experimental group	HFB28	2.90	0.313

**Table 23:** Relative-flexural-strength ratios of the self-healing concrete after exposure to high temperature.

Concrete Group	Flexural Strength after High Tem. (MPa)	Residual Flexural Strength (MPa)	Relative-Flexural-Strength Ratio
Control group	2.86	3.28	1.15
Experimental group	2.61	2.90	1.11

**Table 24:** Results of secondary flexural test of the flexural failure specimens exposed to high temperature after self-healing.

Concrete Group	Designation	Flexural Strength (MPa)	Deflection at Midspan (mm)
Control group	#HFA28	2.02	1.141
Experimental group	#HFB28	1.27	0.719

**Table 25:** Relative-flexural-strength ratios of the flexural failure specimens exposed to high temperature after self-healing.

Concrete Group	Flexural Strength after High Tem. (MPa)	Residual Flexural Strength (MPa)	Relative-Flexural-Strength Ratio
Control group	2.86	2.02	0.71
Experimental group	2.61	1.27	0.49

**Table 26:** Penetration test results of concrete at room temperature.

Concrete Group	Designation	Weight before Water Penetration (g)	Weight after Water Penetration (g)	Water Absorption (%)
Control group	RPA0	1301.3	1344.4	3.3
Experimental group	RPB0	1270.7	1311.9	3.2

**Table 27:** Penetration test results of the concrete after exposure to high temperature.

Concrete Group	Designation	Weight before Water Penetration (g)	Weight after Water Penetration (g)	Water Absorption (%)
Control group	HPA0	1272.0	1403.3	10.3
Experimental group	HPB0	1216.5	1348.8	10.9

**Table 28:** Penetration test results of the self-healing concrete after exposure to high temperature.

Concrete Group	Designation	Weight before Water Penetration (g)	Weight after Water Penetration (g)	Water Absorption (%)
Control group	HPA28	1275.1	1361.2	6.7
Experimental group	HPB28	1230.4	1299.6	5.6

## Data Availability

The data presented in this study are available upon request from the corresponding author.
